# The Necessity for Amending Legislation

**Published:** 1917-04-14

**Authors:** 


### NATIONAL INSURANCE ACT DEVELOPMENTS.

### The Necessity for Amending Legislation.

Thoughtful consideration of the three Reports issued by the Departmental Committee of Inquiry, presided over by Sir Gerald Ryan, and of the Report of the unofficial Commission of Investigation appointed by the Faculty of Insurance, to which reference was recently made in The Hospital, leads to one main conclusion—namely, that amending legislation is urgently required to rectify anomalies in the existing National Insurance Acts, and to secure simplification of detail. If any doubt on the subject should have lingered in the mind after perusal of those Reports, it would assuredly be immediately dispelled upon reference to the Third Report on the work of the National Insurance Audit Department for the year 1916 (Cd. 8488, 2d.). This Report does not specifically refer to the need for such legislation. It merely sets forth statements of facts relating to the general condition of National Insurance accounting as disclosed by the auditors. But these facts are such that there is no escape from the conclusion that the present administrative machinery is too complex and involved to be worked efficiently through the agency of small approved societies, whose secretaries and officials are only able to devote their spare time to the work.

The work which had been done through the agency of these small societies and branches before the passing of the National Insurance Act had been admirable, and it would be impossible to contemplate without serious misgivings the destruction of these voluntary organisations for the encouragement of thrift by straining their capacity beyond the limits of usefulness for which they were brought into existence. The only remedy, therefore, is to bring the requirements of the Act into such a form that they may be workable by societies and branches such as have been indicated.

### Unsatisfactory Accounting.

The Report indicates that on account of the depletion of the audit staff, through the release of men for naval and military service, the scheme of audit had to be substantially modified. In spite, however, of the relaxation in the strictness of the audit, it is an astounding thing to find that out of 18,423 reports on the accounts of approved societies, no less than 10,939, or nearly 60 per cent. of the total, are described as qualified reports, the number of reservations reaching the enormous total of 24,673. This unsatisfactory character of the accounting work is principally confined to the smaller societies and branches, and is attributable in no small degree to the fact that many secretaries of such societies and branches joined the Forces of the Crown, leaving as substitutes persons entirely unacquainted with the details of the work to be done. In these circumstances one feels disposed to make considerable allowance for imperfections in detail, though it is hard to understand why such essential work as the administration of National Health Insurance should not have been considered of sufficient importance to demand that some degree of efficiency should have been shown by substitutes before secretaries were taken away from their work.

### Arrears Regulations a Principal Cause.

The Arrears Regulations are responsible for much of the difficulty that has arisen. In no less than 1,555 cases it became necessary to issue a special report on the failure to make the necessary arrears entries in the contribution registers, while in 11 per cent. of the ordinary reports it was stated that these registers were not properly written up or the arrears calculations were not made or were defective. Failure to make these calculations or to keep the registers properly posted up has the effect of allowing benefit payments to be made in excess of the amount to which the insured persons are entitled. Owing to the widespread default in this particular, and also to the depleted state of the staff, it was not possible for the auditors to disallow the irregular payments of benefit arising from this cause, and no indication is given of the total amount overpaid.

The present Arrears Regulations, therefore, stand out as requiring simplification, with a view to making it possible for an untrained acting secretary of an approved society or branch to carry out the duties of his office without serious risk of involving himself in liabilities for over-payments of benefit.

### Proved Irregularities.

Another serious complaint made by the auditors deals with the delay in the production of branch books for audit. The report refers to repeated applications having to be made for the production of books and accounts, and indicates that the delays to which it refers and of which it gives examples afford opportunities for irregularities.

As to actual defalcations, mention is made of 163 special reports on established irregularities with National Insurance Funds, involving sums amounting to £4,072. Although these items are not large in comparison with the sums disbursed and the numerous officials concerned, yet they compare unfavourably with the corresponding figures of the previous year, and in fact exceed those for the whole of the first three years that the National Insurance Act was in operation. It is possible that some of the defalcations might have been made at an earlier date, and were only brought to light upon investigation of accounts preparatory to a secretary relinquishing his work upon joining the Forces, when he was unable to produce the cash balance shown to be in his hands by his accounts.

The amount is, however, in no way alarming when it is compared with the large total of approximately £9,500,000 disbursed during the year which was the subject of the audit.

Various other causes of complaint are mentioned, as, for instance, the forgery of documents of title in connection with claims for maternity benefit, while committees of management are taken to task for neglecting business-like precautions. Laxity had manifested itself, among other ways, in leaving excessive sums of money under the control of one official; signed blank cheques had also been left in charge of officials, and moneys passed between officers of societies without proper records being kept.

### Simplification is Necessary.

It would, perhaps, be unwise to lay too much stress upon the disclosures contained in this Report, but at the same time they add considerable weight to the reflection that the best endeavours of all who are interested in the mighty system of National Insurance should be used to secure such amendment of the scheme as shall render it more readily understood by the people in general and more simple and direct in its operations. The scheme was overloaded at the outset by endeavouring to appease sectional interests which it has now come to be recognised must be sunk in the common good of the community.

In spite of the urgent calls upon the attention of Parliament, now is the time to press for reform, and not wait for the termination of the war. But quite apart from present relief there is reason to believe that Parliament will agree to any amendments which can be agreed upon by the parties most intimately concerned if they can but arrive at such agreement, by give and take if need be, in an endeavour to secure the common good of all. Those who are most interested are those who have devoted the last few years to strenuous thought and labour on behalf of National Insurance as it is, and who have learned by personal experience where it has failed, and in what directions it can be made to excel. It is to these—the leaders in the administration of approved societies, the officials of Insurance Committees, the Insurance Commissioners and the medical profession—that we must look at the present juncture, and to whom there is a clear call to sink political differences and party interests in a concerted effort to secure the best results from a system that, in spite of all its mistakes, must be admitted to have accomplished a large amount of good.

### Portents of Amending Legislation.

Happily, there are evidences available that matters are beginning to take shape with a view to the introduction of amending legislation. The appointment of Sir Edwin Cornwall as National Insurance Minister has already been accepted as a hopeful augury in this direction. He made it clear early in January that legislation was necessary and desirable, and that it only remained to secure harmony of interest to reach the desired result. We now understand that he has resuscitated the Advisory Committee, which had lain dormant for so long, in a new and more practicable form. As revised it is constituted of those who are generally acknowledged to be the greatest experts in National Insurance matters, and already we learn that its first meeting has been held. National Insurance is a business which must be conducted on business-like lines. The insurance contracts must be clear and intelligible. There must be promptitude in action and thoroughness in organisation. If it is found necessary to spend a little more money per person, even though in total the amount may run into big figures, in order to make the insurance really effective in carrying out the aims and objects of the Act, then that money must be forthcoming. It is useless to disguise the fact that the benefits to-day are only worth in purchasing power approximately one half of what they were when the National Insurance Act was passed in 1911. The benefits were none too large even then, and it would be nothing short of disastrous if the declaration of deficiencies on the first valuation were allowed to be widespread, causing thereby a diminution of benefits.

Moreover, the necessity of conserving infant life, of providing for the mothers, of preventing and curing sickness, and of caring for national health generally, were never greater than they are now.

National Insurance aims at all these objects, and if those whose business it is to look after its administration can but take a wide and statesman-like view and secure a whole-hearted co-operation of all the Health Services, not neglecting the invaluable work of the great voluntary hospitals, then there will be good reason to anticipate the dawn of a new and brighter era for the workers of the nation.

